# Taxing sugar-sweetened beverages: impact on overweight and obesity in Germany

**DOI:** 10.1186/s12889-016-3938-4

**Published:** 2017-01-17

**Authors:** Falk Schwendicke, Michael Stolpe

**Affiliations:** 1 Department of Operative and Preventive Dentistry, Charité - Universitätsmedizin Berlin, Aßmannshauser Str. 4-6, 14197 Berlin, Germany; 2 Kiel Institute for the World Economy, Kiellinie 66, 24105 Kiel, Germany

**Keywords:** Energy consumption, Health economics, Health policy, Obesity, Public health, Tax policy

## Abstract

**Background:**

Consumption of sugar-sweetened beverages (SSBs) increases the risk of overweight and obesity. Taxing SSBs could decrease daily energy consumption and body weight. This model-based study evaluated the impact of a 20% SSB-sales tax on overweight and obesity in the context of Germany.

**Methods:**

The population aged 15–79 years was modelled. Taxation was assumed to affect energy consumption via demand elasticities, which affected weight and BMI. Model-based analysis was performed to estimate the tax impact on BMI in different age, gender and income groups.

**Results:**

Implementing a 20% SSB tax reduced energy consumption mainly in younger age groups, males, and those with low income. Taxation decreased the mean BMI in younger groups, with the largest decrease in those aged 20–29 years, while effects in groups 60 years or above were minimal. In absolute terms, taxation was estimated to avoid 1,028,000 (−3% relative reduction) overweight individuals and 479,000 obese individuals (−4%). Overweight decreased the most in males aged 20–29 years (408,000 fewer cases /−22%), the same applied for obesity (204,000/−22%).

**Conclusions:**

An SSB tax could have significant impact on overweight and obesity, which could translate into substantial reductions of morbidity and mortality.

**Electronic supplementary material:**

The online version of this article (doi:10.1186/s12889-016-3938-4) contains supplementary material, which is available to authorized users.

## Background

Overweight (defined as a body mass index of 25–29.9) and obesity (BMI ≥30) are increasingly common, burdening billions of people around the world [1], while being associated with a large range of diseases spanning from cardiovascular (including hypertension and coronary heart disease) over endodocrinologic diseases (including diabetes mellitus type 2 and hyperlipidemia) to neoplasms and psychologic disorders [2, 3].

Sugar-sweetened beverages (SSBs) are an important dietary energy source. As they are freely available and actively marketed [4], their consumption has been increasing in many countries, contributing to the growing prevalence of obesity [5, 6]. Given that the major SSB consumers are children, adolescents, and (often poorly educated) individuals from lower socio-economic status, who are less aware of SSBs’ harmful effects, there have been calls calling for governmental action to act to reduce SSB consumption [7].

An SSB tax has been suggested to restrict SSB consumption. Given the consumption profile, such tax would likely reduce SSB and energy consumption and associated morbidities mainly in low- instead of high-income groups, thereby alleviating existing inequities in health [8–10]. The revenues of the tax could be further used for further public health actions against overweight and obesity, like subsidizing healthy drinks or foods [4]. SSB taxes are in place in several US states, Mexico and a number of European and Pacific countries [7, 11], and have been found effective for reducing SSB consumption in natural experiments [12, 13].

A number of health economic modeling studies found an SSB tax to reduce the risk of overweight and obesity [4, 14–18]. At present, no evidence on how this tax would reduce overweight and obesity in Germany are available. The present study aimed to provide such evidence.

## Methods

### Overview

**Fig. 1:**
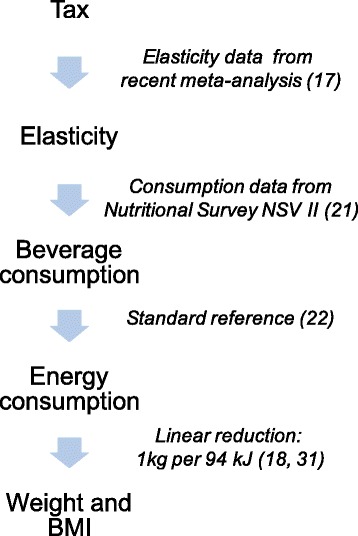
The SSB tax was assumed to affect consumption of different beverages via elasticity of demand, which in turn affected energy consumption and, consequently, body weight and BMI. The references for different data sources are additionally shown

This modelling study was built on the rationale that price increases as a consequence of additional SSB taxation would change SSB and other beverage purchases. The basis for our predictions as to this purchase reaction were empirical price elasticities of demand. Changed purchases resulted in altered energy consumption and, eventually, impacted on an individual’s weight, thereby changing the BMI. We further assumed that beverage consumption and weight/BMI differed according to gender, age and income groups in the II German Nutrition Survey (NSV II). Elasticities were also assumed to differ between income groups. The model underlying this study (Fig. 1) has been previously used to estimate the impact of an SSB tax on dental caries and to estimate potential revenues as well as cost savings from such a tax in the context of German healthcare [19].

### Comparators

Theoretically, an SSB tax can be levied per calorie value or gram of sugar (“specific tax”), or per value of sales unit (“ad valorem tax”), and can be implemented as an excise tax (before sales) or as a sales tax (at point of sale). We modelled the implementation of a national 20% sales tax on SSBs as an addition to existing value added tax (VAT). In our study, SSBs were beverages with added caloric sugars such as lemonades, fruit or sport drinks, but not fruit juice, milk products, non-sweetened (tea or coffee) or artificially sweetened beverages. The comparator was current practice, i.e. 0% SSB tax.

### Setting and perspective

The setting of this study was Germany. As we did not assess any cost of disease impact or tax revenues, no further specifications of the perspective of this study needed to be made.

### Target population and horizon

We simulated the 2015 German population aged 15–79 years. Those aged 14 years or younger were not investigated because consumption data were unavailable, while those aged >79 years were excluded because their SSB consumption was negligible. Modeling was stratified by sex and in 10-year age bands (except for 15–19 years). Population data were drawn from 2012 federal statistics, assuming no changes since then [20]. Household income data were used to construct three income strata, assuming that income was associated with beverage consumption and obesity (as reported in the II German Nutrition Survey, NSV II) as well as own- and cross-price elasticities (using additional data from a recent meta-analysis [17]). As we assumed any changes in energy intake to result in steady state weight changes (see below), we did not need to follow-up patients (i.e. the horizon of this study was a few months until this steady state of weight was reached). Note that this also assumes our price-elasticities explain short-term changes in demand; in the longer term, price elasticities tend to be higher and the effect of the SSB tax on obesity would likely be even larger.

### Beverage and energy consumption

Details on beverage and energy consumption can be found elsewhere [19]. Briefly, estimates of beverage consumption (specifically, SSB, fruit juice, milk) in different gender, age and income groups from the NSV II [21] were matched with population age groups. While the overall volume of consumed beverages was known, the specific consumption of each beverage type was reported only by gender and income. We assumed that relative differences between gender and income groups as to the share of different consumed beverage types per overall volume were constant across age groups, too. This approach was validated by comparing the expected overall consumptions with the summed consumptions of different beverage types in all groups (Table 1). Beverage consumption was transformed into energy consumption via energy density (Table 2) using a Standard Reference [22].

**Table 1:** Consumption of SSBs, juice and milk (ml/day per capita) in different groups

		Male Income groups			Female Income groups		
Age		low	middle	high	low	middle	high
15–18	SSB	416	260	265	416	260	252
	Juice	329	383	333	291	383	372
	Milk	231	231	240	154	154	168
19–24	SSB	690	471	480	306	191	185
	Juice	304	366	318	259	341	331
	Milk	188	188	196	135	135	148
35–34	SSB	517	353	360	198	118	113
	Juice	289	337	293	234	308	296
	Milk	162	162	168	118	118	129
35–50	SSB	302	206	210	150	89	85
	Juice	236	275	239	164	216	207
	Milk	126	126	131	92	92	101
35–50	SSB	178	111	113	63	37	35
	Juice	185	215	172	136	170	162
	Milk	95	95	99	77	77	84
65–80	SSB	59	41	41	41	24	23
	Juice	124	143	114	139	174	165
	Milk	85	85	88	83	83	91

Note that consumption data for water etc. is not given

**Table 2:** Price-elasticities of demand and mean (min/max) energy content of different beverages

	Low or middle income stratum			High income stratum				Energy content (kcal/100 ml)
	**Own**-/Cross-price elasticity							
Beverage	Mean	95% lower CI	95% upper CI	Mean	95% lower CI	95% upper CI	Source	
**SSB**	**−1.21**	**−3.87**	**−0.69**	**−0.908**	**−2.903**	**−0.518**	Long 2015 [17]	42 (37–50)
juice	0.637	0.140	1.447	0.459	0.098	1.0129	Long 2015 [17]	50 (46–56)
milk	0.150	−0.080	0.410	0.188	−0.10	0.513	Long 2015 [17]	60 (55–64)

Own price elasticity is highlighted in bold

Data from a recent meta-analysis [17] were used to estimate elasticities for SSBs (lemonade, fruit drinks), fruit juice and milk (average of whole and skim milk as far as this was assessed). Since elasticity differs by socio-economic (income) status [13, 23], we constructed two elasticity strata (low/middle and high income). Beverage consumption was transformed into energy consumption via energy density using a Standard Reference [22]

### Effectiveness

Our health outcome parameter was the BMI, i.e. an individual’s weight in kg per squared body height in meters. Implementation of an SSB tax was assumed to change energy consumption and, thereby, weight. As body height was stable, BMI changed.

### Price elasticity and pass-on rate

Price elasticity is consumers’ relative increase or decrease in purchases of goods (here: beverages) in reaction to relative price changes (in this case resulting from taxation). Own-price elasticity is the percentage change in purchases of a good when the price of this good changes by one percent; cross-price elasticity is the percentage change in purchases of a good when the price of another good changes by one percent. Since no elasticity data for Germany were obtainable, data reported in a recent meta-analysis [17] were used, details on this can be found elsewhere [19]. Briefly, own- and cross-price elasticities for SSBs (lemonade, fruit drinks), fruit juice and milk (average of whole and skim milk as far as this was assessed) were extracted. Elasticities differ by income status [13, 23], which is why we modelled elasticities in low and middle income groups separately from those in high income groups. Elasticities were assumed to not differ by age or gender (Table 2). As this assumption might lead to distortions via cross-price elasticities (specific age and gender groups who consume only minimal amounts of SSBs are likely to have a lower cross-price elasticity than high-SSB consuming groups), we additionally performed a sensitivity analysis, adjusting cross-elasticities in different age groups for the consumed amount of SSB. This was done by dividing the cross-price elasticities by the average SSB consumption over all age groups in a specific stratum (like male individuals with high income) and multiplying them with the estimated age-group specific SSB consumption, based on the plausible assumption that any consumer’s cross-price elasticity would be zero in the limiting case of zero pre-tax SSB consumption (as there would be no post-tax reduction in SSB consumption to be substituted). That way, cross-price elasticities decreased in (mainly elderly) low SSB consuming groups and increased in (mainly young) high SSB consuming groups.

Manufacturers can pass a tax onto consumers as a price increase. Theoretically and assuming a fully competitive market, this pass-on rate should be 100%, which we modelled in our base-case. However, many markets (the beverage market being one of them) are not fully competitive, with increased costs (as a result of the tax) being possibly absorbed by the manufacturer or even over-shifted to consumers [12, 24–28]. We therefore modelled an 80% pass-on rate in a sensitivity analysis.

### Impact on BMI

To estimate the prevalence of overweight and obesity, data from the most recent national census was used [29], which builds on self-reported data for body weight and height in different sex, age, and income subgroups aged 18 years or above. For those aged 15–17, we used data from the German Study on Children and Adolescents [30]. Both body weight and height were modelled using the gamma function [31].

To estimate changes in BMI per changes in consumed energy, we used published energy balance equations [32], which had been used before for similar modelling studies, assuming that a daily reduction in energy intake of 94 kJ (SD: 2.96) would lead to a body weight reduction of 1 kg [18, 31], with a new steady state body weight established thereafter [33]. We also assumed that the physical activity level of each individual would be unaffected by weight loss.

### Discount rate

Discounting was not applied in this study given the short horizon.

### Analytical methods

Spreadsheet-based Monte Carlo simulations were used to estimate the effect of an SSB tax on body weight and, subsequently, BMI. Parameter uncertainty was introduced by randomly sampling variables from triangular or gamma distributions. Note that parameters were drawn independently, ignoring a possible correlation. For each modeled group, 100 individuals were simulated, each group then being modeled 100 times. Computational constraints prevented a higher number of walks, which might have enhanced the reliability of results. Moreover, confidence intervals were only calculated for the primary analyses (changes in energy intake and BMI), while for population level estimates, only point estimates were reported based on the sum of estimates yielded for separate groups [16]. Microsoft Excel (Microsoft, Redwood, USA), YASAIw (University of Washington) and SPSS 20 (IBM, Armonk, USA) were used for modeling and analysis.

## Results

Based on our simulations, a 20% SSB tax reduced daily energy consumption in males and, to a lesser degree, females (Table 3). The reduction was higher in younger than older people, and in low- than in middle- or high-income individuals. In older individuals, especially females, taxation even increased energy consumption slightly. This was, as the assumed cross-price elasticity led to increased consumption of fruit juices (which have significant caloric value), while SSB consumption decreased only minimally (as absolute consumption was low in this age group even without taxation).

**Table 3:** Mean (SD) change of daily energy consumption (kJ/capita) at 20% SSB tax

Age group	Male income groups			Female income groups		
(years)	Low	Middle	High	Low	Middle	High
15–19	−166 (125)	−65 (93)	−34 (66)	−172 (120)	−68 (87)	−45 (59)
20–29	−376 (179)	−210 (140)	−159 (99)	−128 (81)	−22 (63)	−15 (41)
30–39	−251 (126)	−134 (99)	−119 (80)	−55 (59)	−5 (47)	14 (36)
40–49	−129 (83)	−69 (65)	−54 (48)	−52 (459	5 (38)	5 (25)
50–59	−61 (55)	−9 (38)	−15 (28)	8 (26)	23 (25)	21 (15)
60–69	5 (20)	20 (20)	11 (14)	18 (20)	39 (22)	33 (17)
70–79	4 (21)	21 (20)	11 (26)	19 (24)	38 (21)	31 (16)

Different gender, age and income groups were modelled. Note that in some groups, an SSB tax increased energy consumption, as SSB consumption was minimal even without taxation, but cross-elasticity increased consumption of juice, resulting in higher energy consumption

The mean BMI under current conditions was generally higher in male than female populations (except for those aged 15–19 years), and increased with age up to the age group 60–69 years. BMI was also inversely associated with income. Most individuals aged 30 or above were overweight according to the mean BMI (Table 4). Assuming implementation of an SSB tax, the BMI decreased mainly in younger groups, with the largest decrease in those aged 20–29 years (Table 4). Taxation also decreased the BMI in groups with low rather than high income, and males rather than females. Taxation had no or even minimally disadvantageous effect on BMI in groups 60 years or above (given the described increased consumption of fruit juice).

**Table 4:** Mean (SD) BMI of different gender, age and income groups with and without implementation of an SSB tax

Age group		Male income groups			Female income groups		
(years)		Low	Middle	High	Low	Middle	High
15–19	No tax	24.0 (4.2)	21.7 (2.9)	21.9 (3.2)	24.8 (4.4)	23.7 (3.3)	20.4 (3.5)
	Tax	23.3 (3.9)	21.5 (3.1)	21.7 (3.3)	24.0 (3.9)	23.4 (3.2)	20.2 (3.5)
20–29	No tax	25.2 (3.2)	24.8 (3.9)	23.6 (3.8)	21.4 (3.2)	22.5 (3.9)	21.4 (4.1)
	Tax	23.9 (3.3)	24.1 (3.3)	23.1 (3.5)	21.3 (3.3)	22.5 (4.2)	21.4 (4.1)
30–39	No tax	27.8 (6.1)	25.4 (4.3)	25.8 (4.9)	26.2 (4.3)	23.5 (4.9)	21.8 (5.1)
	Tax	26.9 (5.4)	24.9 (2.9)	25.4 (4.3)	26.0 (4.2)	23.5 (5.5)	21.8 (5.1)
40–49	No tax	28.3 (5.4)	26.9 (6.3)	27.2 (5.6)	25.7 (3.9)	24.7 (5.1)	22.6 (4.6)
	Tax	27.8 (5.8)	26.8 (5.7)	27.0 (5.5)	25.6 (3.6)	24.7 (5.3)	22.6 (4.6)
50–59	No tax	29.1 (5.1	27.6 (5.2)	27.0 (5.3)	27.6 (6.2)	25.2 (6.1)	23.8 (5.1)
	Tax	28.9 (5.9)	27.6 (6.1)	27.0 (4.9)	27.5 (6.4)	25.3 (6.2)	23.7 (4.9)
60–69	No tax	28.6 (6.1)	27.8 (5.8)	27.6 (6.1)	29.6 (6.8)	26.1 (5.9)	25.1 (4.7)
	Tax	28.6 (5.7)	27.9 (5.7)	27.6 (5.7)	29.7 (6.6)	26.2 (5.7)	25.2 (5.0)
70–79	No tax	28.2 (5.5)	26.3 (6.0)	25.9 (4.3)	30.0 (7.1)	26.0 (5.0)	25.2 (5.4)
	Tax	28.2 (6.0)	26.3 (6.1)	26.0 (4.2)	30.1 (7.0)	26.1 (5.5)	25.3 (5.5)

Note that in few groups, assumed cross-elasticity led to increased consumption of juice, while SSB consumption was low anyway; this led to higher BMI if a tax was implemented

On the population level, taxation avoided 1,028,000 (−3%) overweight individuals and 479,000 (−4%) obese individuals. The benefit of a tax for reducing the prevalence of overweight and obesity was largest in those aged 20–29 years and male groups (Table 5). Obesity was also reduced mainly in male populations. Translating these relative changes in the prevalence rates to absolute numbers of avoided cases of overweight and obesity (Table 6) shows that the largest benefit would, again, occur in younger and mainly male groups.

**Table 5:** The relative difference in prevalence rates (in %) of overweight and obesity when levying a 20% SSB tax compared with no tax, in different gender, age and income groups

Age group		Male income groups			Female income groups		
(years)		Low	Middle	High	Low	Middle	High
15–19	Overweight	−13	−9	−8	−12	−9	−7
	Obese	−16	−12	0	−16	−11	0
20–29	Overweight	−28	−20	−20	−20	−7	−5
	Obese	−44	−28	−4	−12	−4	−3
30–39	Overweight	−16	−15	−4	−8	0	+2
	Obese	−16	−14	−8	−8	+2	+3
40–49	Overweight	−4	−4	0	−8	0	+2
	Obese	−4	−2	0	−4	+2	+3
50–59	Overweight	0	0	0	0	+1	+2
	Obese	0	0	0	0	+2	+3
60–69	Overweight	0	+1	0	+1	+2	+3
	Obese	0	+1	0	+2	+2	+2
70–79	Overweight	0	+1	0	0	+2	+5
	Obese	0	+1	0	+2	+4	+7

**Table 6:** Avoided number of overweight or obese individuals (in thousand) per different gender, age and income groups

Age group		Male income groups			Female income groups			Totals
(years)		Low	Middle	High	Low	Middle	High	
15–19	Overweight	16	13	7	28	10	10	84
	Obese	10	4	0	17	4	0	35
20–29	Overweight	140	132	136	80	14	13	515
	Obese	108	80	16	28	6	6	244
30–39	Overweight	108	120	15	44	0	0	287
	Obese	56	60	11	20	0	0	147
40–49	Overweight	36	44	0	60	0	0	140
	Obese	16	16	0	20	0	0	52
50–59	Overweight	2	0	0	0	0	0	2
	Obese	1	0	0	0	0	0	1
60–69	Overweight	0	0	0	0	0	0	0
	Obese	0	0	0	0	0	0	0
70–79	Overweight	0	0	0	0	0	0	0
	Obese	0	0	0	0	0	0	0
Totals	Overweight	302	309	158	212	24	23	
	Obese	191	160	27	85	10	6	

Surprisingly, taxation had no benefit at the population level in most female groups with middle or high income and, generally, in groups aged 60 years or above. Within the applied model, mean energy consumption even increased in these groups (compare with Table 3). That was as absolute consumption levels of SSBs, milk and juice differed markedly in the older compared with younger groups (absolute SSB consumption was very low among the elderly to begin with). Based on age-invariant cross-price elasticities that also did not allow the size of the elasticities to be contingent on initial consumption levels, the same exogenous price change due to a given SSB tax level led to a greater absolute increase in juice consumption among the elderly than their SSB consumption was decreased. Moreover, as both milk and juice were assumed to have higher energy densities than SSBs, this shift in absolute consumption was additionally aggravated when transformed into energy consumption. Last, older age groups generally consumed lower amounts of energy containing beverages (and instead more water), which reduces any impact of an SSB tax in this group.

As one reason for the net-increase in the elderly’s energy consumption from taxation in Table 3 is the implausible invariance of cross-price elasticities with respect to age and initial SSB consumption levels, we additionally applied age-adjusted cross-price elasticities, based on the observation that the elderly have lower SSB consumption before tax and cross-price elasticities would be zero for any person with zero pre-tax SSB consumption, to evaluate the relative changes in the prevalence rates of overweight and obesity (Additional file 1: Table S1). It was notable that except for the age group 15–18 years, the reductions in prevalence were more homogenously distributed across age and income groups, with all groups benefiting from taxation, albeit in some groups at a lower level.

Compared with the base-case, assuming a lower pass-on rate reduced the absolute benefit of taxation (Table 7), as did decreasing the tax rate to 10% or 5%. Applying age-adjusted cross-price elasticities had only limited impact on the overall absolute numbers of avoided overweight or obesity (Table 7).

**Table 7:** Scenario analyses

Scenario	Number of avoided overweight individuals (thousand)	Number of avoided obese individuals (thousand)
Base-case	1,028	479
Pass-on 80%	718	280
Tax 10%	465	164
Tax 5%	276	50
Applying estimated age-adjusted cross-price elasticities	976	449

## Discussion

Based on this study, an SSB tax could decrease calorie consumption and average population BMI in Germany. Such findings are in line with observational studies confirming an effect of a tax on SSB purchases [12, 34]. An SSB tax would be especially beneficial for younger individuals, males, and those with low income (i.e. the main SSB consumers), and could thus be valuable with regard to health equity, too [35]. Future studies should focus on this age group to assess benefits but also potential risks of an SSB tax. Overall and in absolute terms, one could expect a significant number of avoided overweight or obese individuals. The magnitude of the reduction both in relative and absolute terms is also similar to that reported for other countries, assuring us as to the validity of the used model [15–17]. However, when comparing estimated prevalence rates in different groups, we found certain under- and over-estimations in subgroups of income strata (especially for the prevalence for obesity), while the overall estimate (regardless of income groups) was near congruent with those found in epidemiologic surveys [30, 36].

Based on our findings, it is likely that the group to benefit the most are those aged 20–29 or, more generally, younger individuals, with higher benefits in male than female. This was mainly due to SSB consumption being high in these groups, while juice and milk consumption were low. It should be highlighted that, as discussed, it is uncertain if there would truly be an overall increase in energy consumption in elder and female groups, as our results in Table 3 suggest. Among the elderly, relatively stable behavioral patterns might have formed, which would mean that their own- and cross-price elasticities are lower than estimated for all age groups combined. Moreover, cross-price elasticities are likely to be lower at low initial levels of SSB consumption, as observed among the elderly. Applying different cross-price elasticities in low SSB consuming groups adjusted for the possible distortion and found the benefits of the tax to be more homogeneously distributed. However, as these age-specific estimates of elasticities were constructed on the basis of plausible behavioral assumptions instead of empirical evidence (to assess the robustness of our findings rather than to estimate precise effects), caution is needed when interpreting the findings of this sensitivity analysis.

This study has a number of strengths and limitations. First, nationally representative consumption data was used, which increases confidence in the policy implications. However, NSV II used dietary recording via diaries for estimating beverage consumption, which is prone for bias by misreporting. Further studies should strive and validate these consumption estimates using sales data. Data from NSV II could also be regarded as potentially outdated, as they were more than 7 years old; given the historic secular trend of an increasing consumption [7, 37] we could have under-estimated the effects of a tax on energy consumption and weight. Similarly, weight and height estimates were self-reported, with possible bias leading to underestimation of the BMI and possible underestimation of the benefits of a tax.

Second, elasticities were derived from a number of sources, as reported in a recent meta-analysis, and might not fully apply to Germany, leading to some distortions. Moreover, the used elasticity estimates are not age-specific and may thus lead to an over-estimation of the increase in juice consumption that an SSB tax triggers among the elderly, an issue which requires further study on the basis of age-specific elasticities. Ideally, one should also allow elasticity estimates to vary with initial SSB consumption levels (as attempted in our sensitivity analysis).

Third, the very concept of price elasticity of demand is an inevitable simplification based on the assumption that observed market demand is informative of individual behavioural responses to exogenous price changes, raising the issue of aggregation. Yet in research on tax policy, price elasticity is a well-established analytical tool to characterize (in one precise and easily compared summary measure) the shape of demand curves, i.e. plots of the geometric location of observed demand in a diagram with given prices of a good on the vertical axis and the quantities demanded at these prices on the horizontal axis, and to predict the empirical incidence of new taxes or tax changes. While in economic theory it is possible to specify downward-sloping demand curves by means of mathematical functions with constant price elasticity along the entire curve, the exact shape of real-world demand curves is usually unknown and could only be estimated with a very large number of individual observations. Their price elasticity is likely to vary with the initial level of demand before an exogenous price change, such as a tax, is introduced so that any elasticity estimate can only be an approximation for the relevant initial price level. An additional caveat is that price elasticities tend to ignore income effects of price changes, i.e. the reduction in available income for other goods after a purchase is made, but these effects are likely to be negligible in the present context as spending on SSBs is likely to be only a small proportion of consumers’ total spending on consumption goods.

Fourth, substitution of SSBs with sugary foods has also not been modelled but might have some impact on energy consumption; there might even be a complementary relationship, implying an even larger benefit from taxing SSBs [38]. Fifth, the association between energy consumption and weight was assumed to be linear, leading to a new steady state after weight reduction occurred. This assumption is certainly a simplification, as different equations for men and women are likely to apply [15, 16, 39]. Metabolic rates might change after weight loss (for example due to different mobility of lighter than heavier individuals). Moreover, changes in energy intake due to elasticity might take place over a long-term period, which we did not account for. Similarly, we did not draw parameters from a correlation matrix, while weight, height and SSB consumption are likely to be correlated. Last, our study only describes the effects of a tax on obesity, while a range of other diseases may be triggered or aggravated. That, in turn, would also affect the cost-effectiveness. In general, it would be relevant to assess not only the effects of sugar consumption via increased energy intake, but also other proposed harmful effects of sugary diets [40].

## Conclusions

Within these limitations and the specific assumptions made in this study, implementing a 20% tax on SSBs is likely to reduce overweight and obesity mainly in younger age groups, males and those with lower income. This finding, however, is highly dependent on estimates of the underlying own-price and cross-price elasticities, which might be improved through future research. In general, we expect significant relative and absolute benefits, which could translate into relevant effects on morbidity and mortality.

## Additional file

Additional file 1: Table S1. Sensitivity analysis. The relative difference in prevalence of overweight and obesity rates when levying a 20% SSB-tax compared with no tax, when applying age-adjusted cross-price elasticities (in %). Compare with Table 5, where cross-price elasticities were not age-adjusted. (DOC 39 kb)

## Abbreviations

BMI: Body mass index
SSB: Sugar sweetened beverages
VAT: Value added tax
