# Supplementary material for: Taxing sugar-sweetened beverages: impact on overweight and obesity in Germany
Source: BMC Public Health. 2017 Jan 17;17:88. doi: 10.1186/s12889-016-3938-4 (PMC5240244; doi:10.1186/s12889-016-3938-4)
Supplement: Additional file 1: Table S1. — Sensitivity analysis. The relative difference in prevalence of overweight and obesity rates when levying a 20% SSB-tax compared with no tax, when applying age-adjusted cross-price elasticities (in %). Compare with Table 5, where cross-price elasticities were not age-adjusted. (DOC 39 kb) [file 12889_2016_3938_MOESM1_ESM.doc]

Table S1 Sensitivity analysis. The relative difference in prevalence of overweight and obesity rates when levying a 20% SSB-tax compared with no tax, when applying age-adjusted cross-price elasticities (in %). Compare with Table 5, where cross-price elasticities were not age-adjusted.

| Age group |  | Male income groups | | | | Female income groups | | | |  |
| --- | --- | --- | --- | --- | --- | --- | --- | --- | --- | --- |
| (years) |  | low | middle | high | low | | middle | high |  | |
| 15–19 | Overweight | -12 | -10 | -7 | -11 | | -10 | -6 |  | |
|  | Obese | -15 | -11 | -9 | -12 | | -10 | -4 |  | |
| 20–29 | Overweight | -15 | -15 | -9 | -10 | | -7 | -4 |  | |
|  | Obese | -24 | -19 | 0 | -8 | | -5 | -3 |  | |
| 30–39 | Overweight | -6 | -8 | 0 | -5 | | -2 | -1 |  | |
|  | Obese | -13 | -12 | -6 | -12 | | -11 | -1 |  | |
| 40–49 | Overweight | -4 | 0 | 2 | -14 | | -4 | -1 |  | |
|  | Obese | -2 | -1 | -2 | -6 | | -7 | -2 |  | |
| 50–59 | Overweight | -4 | 0 | -3 | -1 | | -1 | -2 |  | |
|  | Obese | -7 | -15 | -3 | -1 | | -4 | -2 |  | |
| 60–69 | Overweight | -7 | -0 | -3 | -1 | | -1 | -2 |  | |
|  | Obese | -1 | -1 | -1 | 0 | | -1 | 1 |  | |
| 70–79 | Overweight | -4 | 0 | -2 | -1 | | -1 | 0 |  | |
|  | Obese | -7 | -1 | 0 | -2 | | -7 | -1 |  | |
